# Ultrasound plays a key role in imaging and management of genital angiomyofibroblastoma: a case report

**DOI:** 10.1186/s13256-015-0715-4

**Published:** 2015-10-28

**Authors:** Benjamin Wolf, Lars-Christian Horn, Romy Handzel, Jens Einenkel

**Affiliations:** 1 Department of Obstetrics and Gynecology, Leipzig University, Liebigstrasse 20a, 04103 Leipzig, Germany; 2 Institute of Pathology, Leipzig University, Liebigstrasse 24, 04103 Leipzig, Germany

**Keywords:** Angiomyofibroblastoma, Transvaginal ultrasound, Mesenchymal tumors of the lower genital tract

## Abstract

**Introduction:**

Angiomyofibroblastoma is a benign, rare mesenchymal tumor arising from the genital tract of both men and women and was first described by Fletcher and colleagues in 1992. The tumor needs to be distinguished from other, similar lesions, such as deep and superficial aggressive angiomyxoma and cellular angiofibroma, because aggressive angiomyxoma demands much more extensive treatment. The vast majority of angiomyofibroblastomas arise from the vulva and appear as solid cystic masses on ultrasound images.

**Case presentation:**

We report a case of a 35-year-old Caucasian woman with an angiomyofibroblastoma arising from the vagina. She presented with a painless mass of about 5cm in diameter that had a rather homogeneous, hypoechoic appearance on ultrasound images. The patient underwent surgical resection of the mass, which was subsequently diagnosed as angiomyofibroblastoma. We present sonographic and magnetic resonance imaging findings, intraoperative and histologic images, and a thorough review of the literature.

**Conclusions:**

In our opinion, ultrasonography is the most valuable tool to establish a preoperative diagnosis of this tumor entity, differentiate it from other lesions of the female genital tract, and plan surgery accordingly. Even though it is a rare tumor, gynecologists should be able to recognize it and to differentiate it from other tumor entities that demand more aggressive treatment. We describe a different sonographic appearance of this tumor than previously reported.

**Electronic supplementary material:**

The online version of this article (doi:10.1186/s13256-015-0715-4) contains supplementary material, which is available to authorized users.

## Introduction

Angiomyofibroblastoma (AMFB) was first described by Fletcher and colleagues in 1992 as a distinct subgroup of mesenchymal tumors histologically similar to aggressive angiomyoma (AA) that usually arise from the genital tract in both men and women [1]. The vast majority of cases originate from the vulva (Fig. 4a). One case of AMFB in the nasal cavity has been described recently [2].

In contrast to AA, which is characterized by local destructive growth and recurrence after clear margin resection in up to 47% of cases [3–5], AMFB has an excellent prognosis. Clinically, the tumor has well-circumscribed margins, a size of usually about 5cm at the time of diagnosis, and virtually no tendency for local recurrence after excision. Histologically, these tumors are characterized as being composed of plump, ovoid, or, less often, spindle-shaped cells, with limited eosinophilic cytoplasm and ovoid nuclei [1, 6]. Characteristically, the stroma contains many capillary-sized blood vessels, around which the characteristic cells aggregate [6].

Together with cellular angiofibroma (CA) and myofibroblastoma (MFB), AMFB belongs to a group of benign stromal tumors of the lower female genital tract. These tumors, along with AA, have significant overlap in their morphological and immunohistochemical characteristics, which can pose serious diagnostic problems [7]. Recent research, however, has shown that a common cytogenetic aberration present in angiofibroma (AF) and MFB (loss of the 13q14 region) is not shared by AMFB, suggesting that AMFB is not genetically related to AF and MFB [7].

## Case presentation

A 35-year-old Caucasian woman was referred to our outpatient clinic for evaluation of a neoplastic lesion located adjacent to the uterine cervix. She reported not having experienced any pain or discomfort with regard to the lesion and that she had not noticed any changes in bladder or bowel function. The lesion had come to her gynecologist’s attention during a routine pelvic examination 1 week earlier. Her history was remarkable for the excision of a melanoma from her right inner thigh about 4 years earlier. Two inguinal lymph nodes that were excised for sentinel staging at the time were reportedly without metastasis.

In her gynecological examination at our clinic, no inguinal lymph node swelling was noted, and her vulva and proximal vagina appeared normal. The vaginal mucosa on the right side of the posterior fornix was bulging inward, displacing the uterine cervix laterally to the left. Underlying the mucosa was a palpable, semimobile mass of about 5cm in diameter that was of plump, elastic consistency. The vaginal mucosa overlying the tumor was smooth but not mobile relative to the tumor. There were no signs of ulceration or retraction by the tumor.

**Fig. 1:**
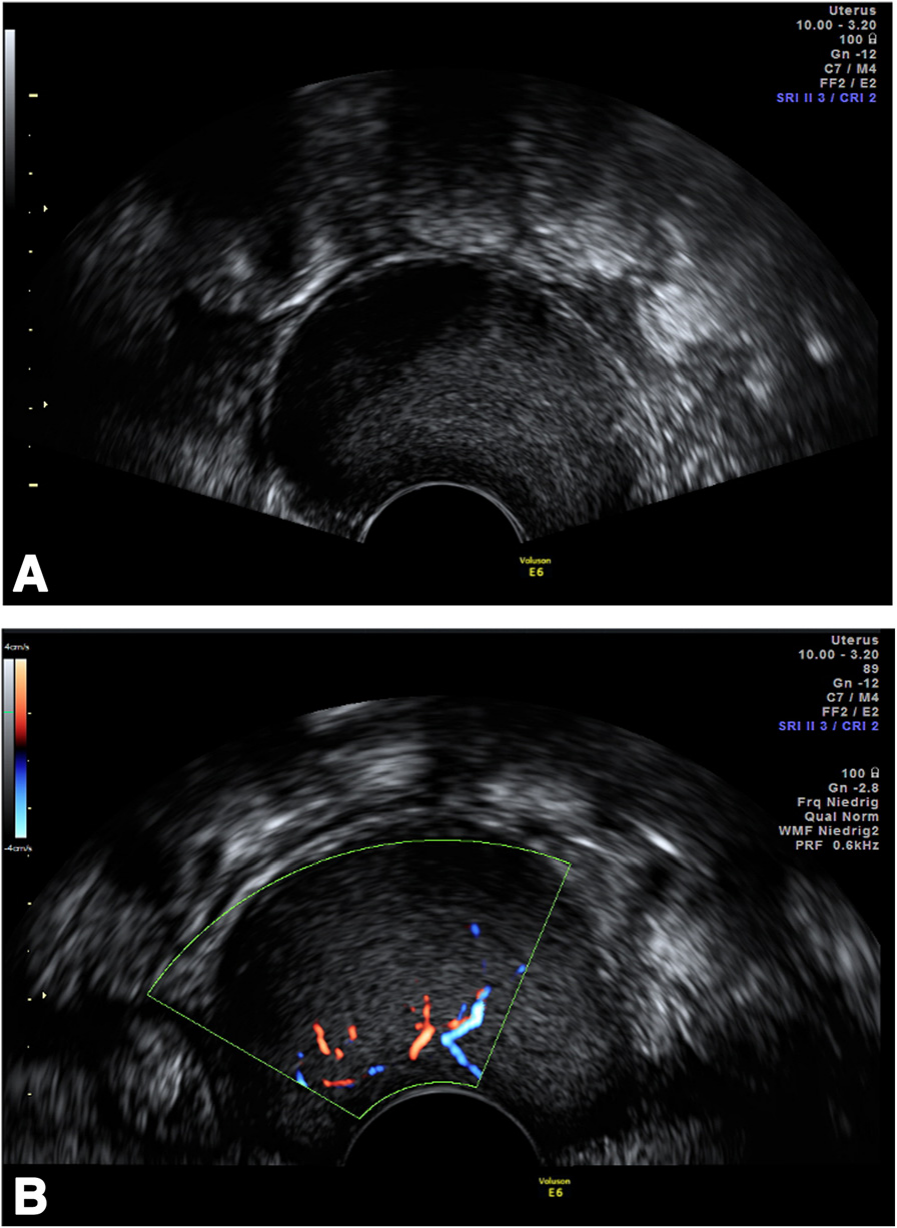
**a** Transvaginal ultrasound image shows a well-demarcated, homogeneous mass of medium echogenicity (sagittal view). Intralesional septations can be seen only in Additional file 1. **b** Color Doppler imaging reveals intralesional vascularization

**Fig. 2:**
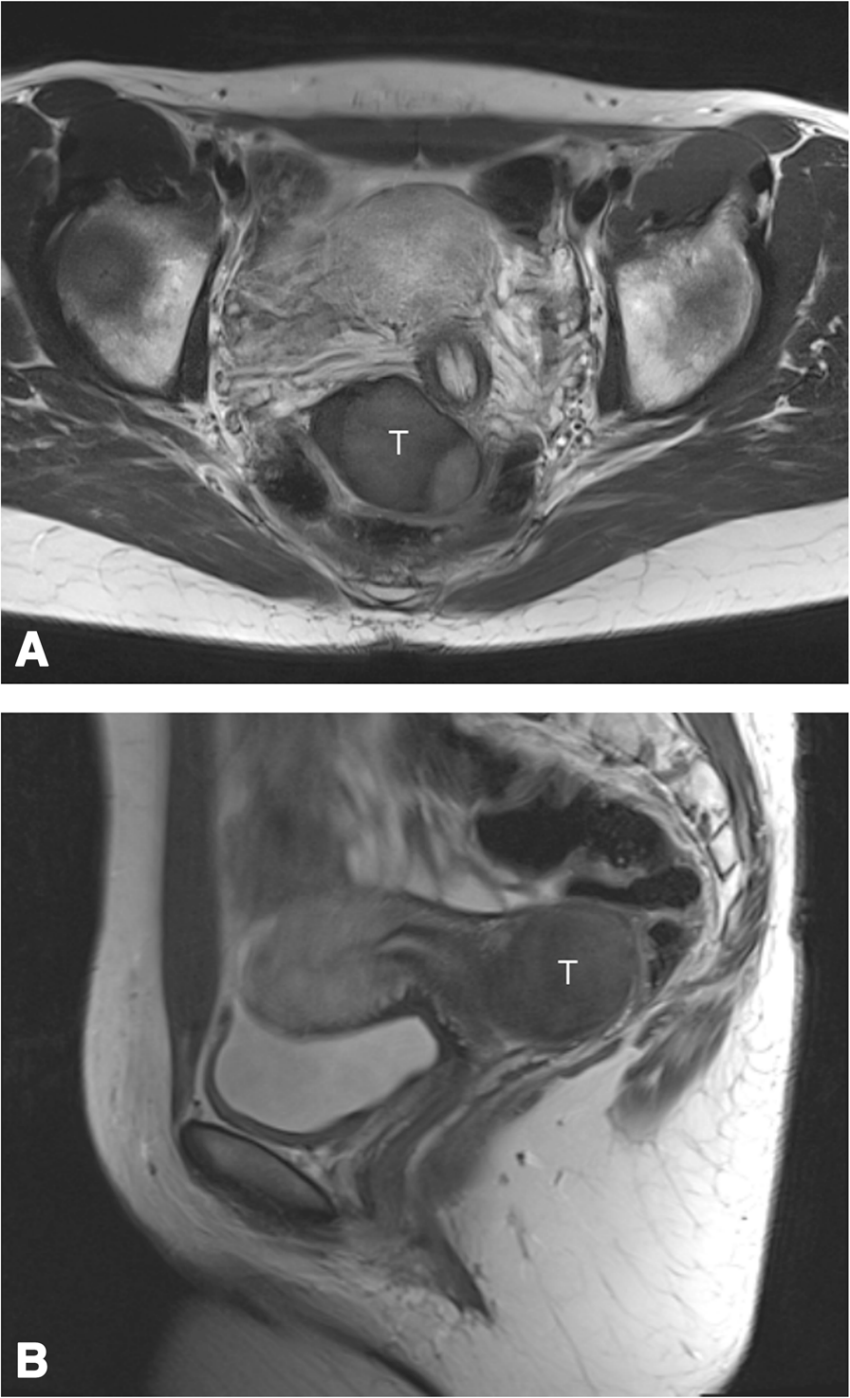
Coronal (**a**) and sagittal (**b**) T2-weighted magnetic resonance images obtained preoperatively show a homogeneous solid mass (T) displacing the cervix at *left* (seen best in coronal view)

**Fig. 3:**
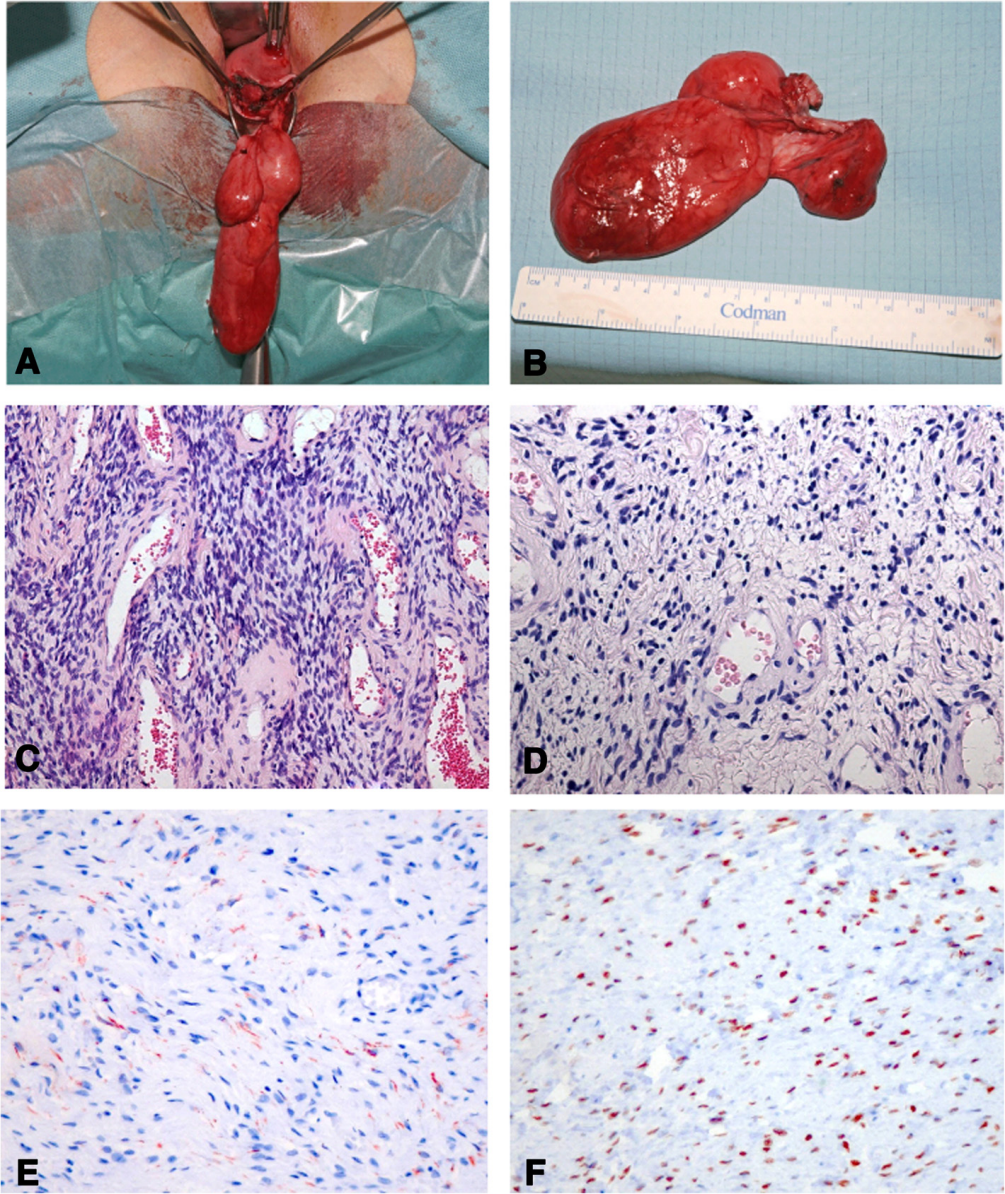
**a** and **b** Intraoperative appearance of the tumor. The tumor was covered entirely with a frail membrane of pinkish gray appearance. **c** Cellular mesenchymal lesion with alternating cellularity intermingled with small blood vessels [hematoxylin and eosin (H&E) stain, original magnification ×109]. **d** Higher-magnification image representing thin-walled blood vessels surrounded by ovoid to spindle-shaped cells with some epithelioid appearance and abundant eosinophilic cytoplasm (H&E stain, original magnification ×241). **e **Immunohistochemical staining for desmin with weak positivity. **f** Strong nuclear expression of estrogen receptor.

Sonographically, the mass appeared homogeneous with medium echogenicity, few septations, and smooth edges (Fig. 1), and it had no papillary projections. Color Doppler imaging revealed several vessels visible within the structure. Subsequently, we took a core biopsy specimen of the tumor while the patient was under general anesthesia. A pathological examination revealed a mesenchymal tumor, most probably benign owing to the absence of any mitotic figures; however, it was not possible to classify the tumor any further. We discussed the findings with the patient and recommended surgical excision of the tumor. Owing to the unusual location of the tumor, we obtained additional magnetic resonance (MR) images of the pelvis (Fig. 2). T1- and T2-weighted images showed a homogeneously hypointense lesion that led to deviation of the cervix and the rectum. For excision, we performed a horizontal colpotomy about 1.5cm distant from the cervix, including the biopsy canal. The tumor appeared encapsulated and was dissected from the surrounding tissue. Gross examination of the tumor showed a 9×6×1.5-cm pinkish gray mass weighing 65g. The tumor was covered entirely with a frail, well-vascularized membrane (Fig. 3a).

Histologically, the highly cellular lesion represented varying cellularity with vascularization by thin-walled blood vessels surrounded by spindle-shaped epithelioid cells with abundant eosinophilic cytoplasm and positive immunostaining for desmin and estrogen receptor (Fig. 3c, d).

The mesenchymal cells showed a diffusely positive reaction to CD34, and 70% stained positive for desmin. There was no staining reaction to smooth muscle actin and S100. About 90% of the cells were estrogen receptor–positive. On the basis of these characteristics, a diagnosis of AMFB was made.

At her follow-up examination 17 months postoperatively, the patient was doing well and without any evidence of recurrent disease or sexual or urinary dysfunction.

## Discussion

**Fig. 4:**
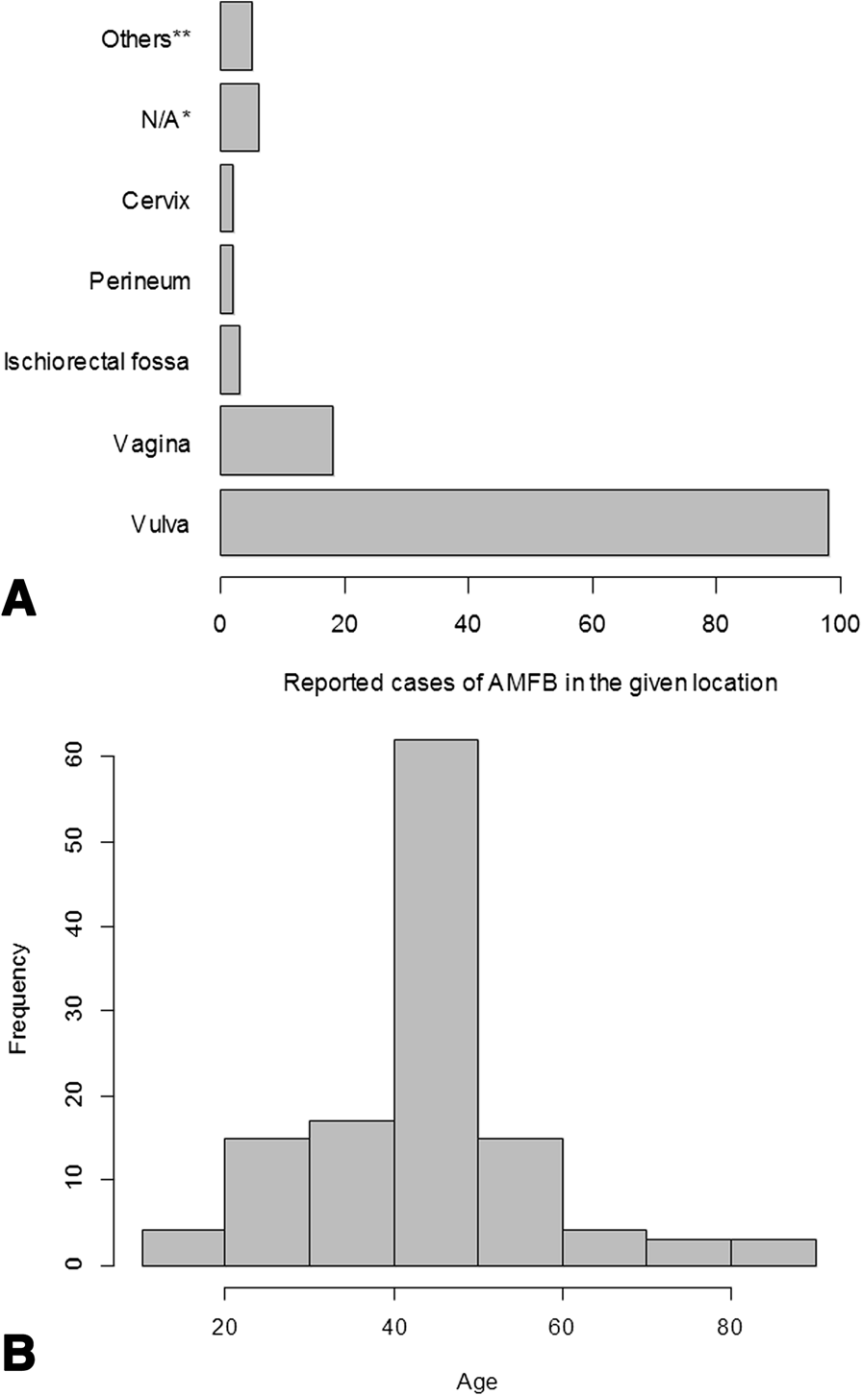
Analysis of all 125 cases of angiomyofibroblastoma in women reported to date (see also Additional file 2). **a** The number of cases of angiomyofibroblastoma occurring at each given location out of a total of 125 cases is shown. *N/A denotes that no information regarding the location was available. **Other locations include inguinal, urethral, fallopian tube, perianal, cul-de-sac, and retroperitoneal. **b** Histogram showing the age distribution of 116 patients with angiomyofibroblastoma (including men). For 21 patients, no age information was available for analysis. *AMFB* angiomyofibroblastoma

Including our patient, 137 cases of AMFB have been reported to date (for a detailed list, see Additional file 2). The median age of all patients at the time of presentation was 45 years (Fig. 4b), the majority of the patients had been aware of their tumors for about 1 year. No recurrences have been reported after excision, although it has to be pointed out that the median follow-up of all cases published thus far is only 12 months. (Information about follow-up was available for only 64% of published case reports; see Additional file 2). Only one case of sarcomatous transformation has been reported; however, there was no recurrence after resection in that patient [8]. We found the female-to-male ratio to be 10:1. In women, the vast majority of tumors are located in the vulva (Fig. 4a). Owing to their low frequency, AMFBs and other vaginal soft tissue tumors can easily be confused clinically or sonographically with other, more common vaginal masses, such as Bartholin’s cyst, rectocele, or urethral diverticulum [6, 9–11]. However, soft tissue tumors can be differentiated from these masses by demonstrating intralesional vascularization using color Doppler imaging. Once this has been accomplished, echogenicity and demarcation of the tumor can help to further establish the diagnosis.

Sonographically, AMFB has been reported to be well demarcated with inhomogeneous echogenicity and multiple hypoechoic areas within an echogenic stroma [12]. Wang and coworkers assessed 72 perineal tumors, two among which represented AMFB and were characterized as solid cystic masses on the basis of ultrasonography [13]. In contrast to these descriptions, we found the tumor in our patient to be of homogeneous, medium echogenicity without solid cystic features (see Additional file 1). Retrospectively, the obtained MR images did not add much information to what was already known based on the ultrasound studies.

To date, the exact pathogenesis of AMFB is not clear. Because many tumors express estrogen and progesterone receptors, it is likely that these hormones play a crucial role in the pathogenesis of AMFB. Indeed, these tumors almost exclusively occur in women of reproductive age; two cases of postmenopausal women receiving tamoxifen therapy have been reported [14, 15]. Because many of the histological and immunohistochemical features of AMFB, AA, and CA overlap, it has been suggested that these richly vascularized myxoid tumors of the genital tract represent members of the same fibroblastic–myofibroblastic tumor spectrum [16]. Sonography is a widely available, relatively cheap, extremely valuable tool to characterize and distinguish these tumors preoperatively and to plan surgery accordingly. In our opinion, MR imaging is not mandatory in the evaluation of soft tissue lesions of the lower genital tract such as AMFB in women. Postoperative pathological examination of the surgical specimen remains the only way to definitively establish a diagnosis of AMFB.

## Conclusions

AMFB is a rare, benign stromal tumor arising most commonly from the female lower reproductive tract, and ultrasonography is the most important imaging modality in its preoperative diagnosis and management. AMFB appears as a homogeneous, well-defined, vascularized lesion of medium echogenicity. AMFB needs to be distinguished from aggressive angiomyxoma because the latter demands more extensive treatment.

## Consent

Written informed consent was obtained from the patient for publication of this case report and any accompanying images. A copy of the written consent is available for review by the Editor-in-Chief of this journal.

## Additional files

Additional file 1: **Ultrasonographic appearance of AMFB.** Collection of short ultrasound clips showing the characteristics of AMFB. (WMV 11397 kb)

Additional file 2: **Published case reports of AMFB up to the time of the writing of this report.** (XLSX 21 kb)

## Abbreviations

AA: aggressive angiomyoma
AF: angiofibroma
AMFB: Angiomyofibroblastoma
CA: cellular angiofibroma
H&E: hematoxylin and eosin
MFB: myofibroblastoma
MR: magnetic resonance
